# Supplementary figures and images for: Prevalence and associated factors of physical-psychological-cognitive multimorbidity in Chinese community-dwelling older adults: a cross-sectional study
Source: PeerJ. 2025 Jul 24;13:e19750. doi: 10.7717/peerj.19750 (PMC12296576; doi:10.7717/peerj.19750)

Supplementary figure 1: Participant Recruitment Flow Diagram for the Study.

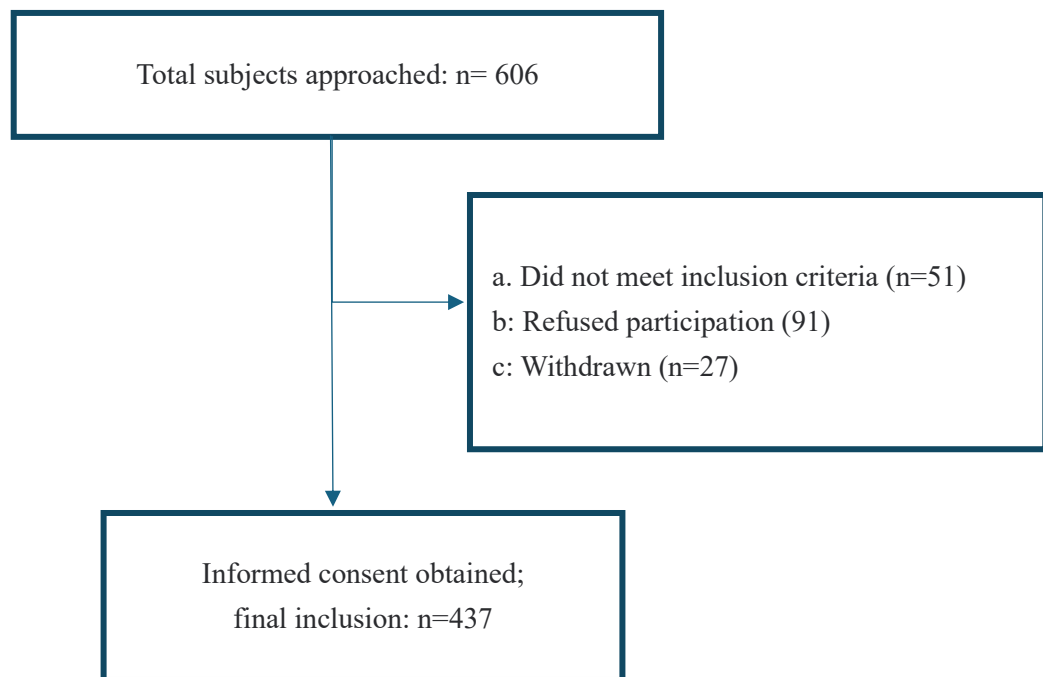

Supplement: Supplemental Information 2 [file peerj-13-19750-s002.pdf]
