# Supplementary material for: Prevalence and associated factors of physical-psychological-cognitive multimorbidity in Chinese community-dwelling older adults: a cross-sectional study
Source: PeerJ. 2025 Jul 24;13:e19750. doi: 10.7717/peerj.19750 (PMC12296576; doi:10.7717/peerj.19750)
Supplement: Supplemental Information 3 [file peerj-13-19750-s003.docx]

| **Supplementary table 1 The Chinese utility value set for the EQ-5D-5L health states** | | | | | |
| --- | --- | --- | --- | --- | --- |
| Level | MO | SC | UA | PD | AD |
| 1 | 0 | 0 | 0 | 0 | 0 |
| 2 | 0.066 | 0.048 | 0.045 | 0.058 | 0.049 |
| 3 | 0.158 | 0.116 | 0.107 | 0.138 | 0.118 |
| 4 | 0.287 | 0.210 | 0.194 | 0.252 | 0.215 |
| 5 | 0.345 | 0.253 | 0.233 | 0.302 | 0.258 |
| MO: Mobility; SC: Self-care; UA: Uaual activities; PD: Pain/Discomfort; AD: Anxiety/Depression. | | | | | |
